# The Relationship Between Humor and Subjective Well-Being: The Mechanism of the Connotation of Humor

**DOI:** 10.3390/bs16030382

**Published:** 2026-03-06

**Authors:** Yamin Cai, Ji Zhang, Man Leng, Qingli Guan, Xiaodong Zhou, Song Zhou, Wenbo Zhou

**Affiliations:** 1 Department of Mechanical and Materials Engineering, Changzhou University Huaide College, Taizhou 214500, China; 2 School of Psychology, Fujian Normal University, Fuzhou 350117, China; 3 Department of Psychology, University of Chinese Academy of Sciences, Beijing 101408, China; 4 School of Human Sciences, University of Western Australia, Perth 6009, Australia

**Keywords:** subjective well-being, humor, perspective-taking, emotional intelligence

## Abstract

Humor has emerged as a vital component of human life, significantly influencing individuals’ well-being. This study aims to explore the effect of sense of humor on individuals’ subjective well-being and the mediating roles of perspective-taking and emotional intelligence. A total of 1007 Chinese participants (*M_age_* = 23.79, *SD* = 7.95) were recruited. The results showed that sense of humor, as well as the four dimensions of humor, positively affects individuals’ subjective well-being. Perspective-taking and emotional intelligence play roles as chain mediators between humor and subjective well-being. Moreover, the direct effects of sense of humor, attitude towards humor, humor production, and coping humor on subjective well-being are significant. However, the direct effect of humor appreciation on subjective well-being is not significant. Altogether, this study demonstrates how sense of humor, perspective-taking, and emotional intelligence contributes to subjective well-being.

## 1. Introduction

The rise of short video platforms has led many social media users to employ humor to capture attention (Li, 2022), sparking criticism of an emerging “pan entertainment” culture. Yet research shows that entertainment can also yield positive outcomes, with humor serving as a key medium in interpersonal exchange (Taylor et al., 2025). People use humor not merely for fun but as an effective coping strategy (Nielsen et al., 2024). In contemporary society, individuals increasingly focus on inner experiences, making subjective well-being a central indicator of mental health (Hansen et al., 2023). While external circumstances matter, internal psychological mechanisms are equally vital; humor, in particular, impacts subjective well-being through multiple pathways (Jiang et al., 2020; Papousek, 2018; Santhosh & Appu, 2015). Beyond providing pleasure, humor fulfills social functions—emotional regulation, stress relief, and relationship enhancement! (Cao et al., 2023; Karami et al., 2018; W. F. Ruch et al., 2018).

Existing studies confirm humor’s role in boosting well-being by fostering social ties and stress coping (Amjad & Dasti, 2022; L. Zhu et al., 2023), yet they overlook the specific pathways, especially the contributions of perspective-taking and emotional intelligence. Exploring these mediators can deepen our understanding of emotion and social interaction. Moreover, humor research has largely compared distinct humor styles, leaving its internal structure underexamined (Kokkinos & Koutsospyros, 2023). This study moves beyond simple style comparisons to investigate humor’s multidimensional facets, aiming to clarify its complex effects on subjective well-being, expand theoretical frameworks, and offer fresh insights into their interplay.

### 1.1. Sense of Humor and Subjective Well-Being

Subjective well-being is a multifaceted construct defined as an individual’s comprehensive assessment of life quality, shaped by personal criteria. This evaluation typically involves the prevalence of positive affective states, the relative absence of negative emotions, and a deep sense of life satisfaction (Diener et al., 2003). To systematically measure subjective well-being, researchers often categorize it into key indicators grouped into three main aspects: cognitive evaluation, reflecting an individual’s overall life satisfaction; positive affect, capturing emotional experiences such as happiness; and negative emotions, including anxiety, depression, sadness, loneliness, boredom, discomfort, and other emotional experiences, but excluding major emotional disorders and neurosis (Chen & Zhou, 2003). Among the diverse factors influencing subjective well-being (He et al., 2025), an individual’s sense of humor is particularly noteworthy (Zulfadri & Raudatussalamah, 2019). Humor refers to the differences in habitual behaviors, emotional responses, attitudes, and cognitive abilities that individuals demonstrate during humorous interactions (W. Ruch, 2010). This construct integrates cognitive, emotional, and behavioral elements, each playing a critical role in the processes of humor perception, comprehension, appreciation, and production (W. Ruch, 2010). To further elucidate this concept, four distinct dimensions of humor were identified (Thorson & Powell, 1993). These dimensions include attitude towards humor, reflecting an individual’s general outlook on humor and humorous individuals; humor production, including both the creation and social use of humor; coping humor, referring to the use of humor as a strategy for managing stress; and humor appreciation, involving the cognitive ability to perceive and evaluate humor. These dimensions provide a comprehensive framework for examining the impact of humor on subjective well-being.

The hedonic treadmill model suggests that individuals experience a temporary surge in positive emotions when they achieve goals or encounter positive events, though this emotional boost tends to diminish over time (Diener et al., 2006). To counteract this adaptation and sustain subjective well-being, individuals require stable psychological resources. A sense of humor fulfills this critical role, functioning not as a fleeting emotional reaction but as an enduring personality trait that provides a continuous stream of positive affect to counteract hedonic adaptation, thereby sustaining subjective well-being (W. Ruch, 2010). Specifically, this sustaining effect is achieved through two distinct pathways. First, as a coping mechanism, humor enables individuals to manage adversity and stressful situations more effectively, fostering psychological resilience and adaptability, which in turn enhances life satisfaction (R. A. Martin & Ford, 2018). Second, humor facilitates interpersonal relationships by helping individuals establish and maintain connections or resolve conflicts, contributing to smoother social interactions and greater life satisfaction (Kim et al., 2022). Thus, by facilitating adaptive coping and fostering social connectivity, sense of humor functions not merely as a reaction to happiness but as a stable trait that actively promotes and sustains subjective well-being (R. Martin & Kuiper, 2016; Veenhoven, 2012). Beyond enhancing well-being, humor also serves a protective function by mitigating well-being by alleviating stress (Froehlich et al., 2021), reducing test anxiety (Ford et al., 2012), and curbing aggressive behavior (H. Zhu et al., 2022). Empirical studies consistently support this link between humor and subjective well-being. For instance, it was found that individuals who frequently employ positive humor styles report higher levels of well-being (Ford et al., 2016). Similar results have been observed across cultural contexts, including China, where humor was found to positively predict subjective well-being (Yue et al., 2016). Based on this theoretical foundation and empirical evidence, we hypothesize that sense of humor acts as a significant positive predictor of subjective well-being (H1).

### 1.2. Perspective-Taking and Emotional Intelligence

Emotional intelligence (EI) encompasses the capacity to recognize and comprehend emotional states in oneself and others and to use this awareness to manage behavior and relationships (Goleman, 1998). From a functional perspective, sense of humor is intrinsically linked to these EI competencies. The effective use of humor is not merely a linguistic skill but a complex emotional process that actively exercises and refines emotional sensitivity and regulation (Huang & Lee, 2019). Specifically, the influence of humor on emotional intelligence unfolds across two distinct dimensions: social perception regarding others and emotional management regarding the self. First, regarding social perception, individuals with a high sense of humor demonstrate a profound comprehension of others’ emotional landscapes, allowing them to detect subtle emotional cues and deliver humor appropriately (Vernon et al., 2009). Second, regarding self-regulation, humor serves as a sophisticated coping mechanism that requires individuals to continuously monitor their own emotional states and cognitive appraisal processes to effectively confront adverse circumstances (Wang et al., 2019). Consequently, the active practice of humor fosters the development and reinforcement of the core skills of emotional intelligence.

In turn, this heightened emotional intelligence serves as a robust predictor of subjective well-being (Pan et al., 2022). This occurs through two pathways: internally, high-EI individuals are adept at transforming negative moods into positive ones, maintaining an emotional equilibrium conducive to psychological health (Troth et al., 2023); externally, their heightened awareness of others’ emotions facilitates the cultivation of harmonious social relationships, which are essential for life satisfaction (Wollny et al., 2020). Synthesizing these relationships, we propose that sense of humor facilitates subjective well-being by activating and enhancing emotional intelligence capabilities. Humor augments an individual’s capacity to comprehend and regulate emotion, which subsequently fosters adaptive coping and favorable social interactions, ultimately contributing to well-being (Amjad & Dasti, 2022; Kim et al., 2022). Accordingly, we posit the following hypothesis (H2): Emotional intelligence serves as a mediating factor in the relationship between sense of humor and subjective well-being.

Perspective-taking is considered an individual’s cognitive process of concluding others’ viewpoints and attitudes as precisely as possible in specific situations (Epley et al., 2006). It is a complex cognitive task; individuals should extract the required information from multiple sources and employ various strategies to infer the mental states of others. The predictive effect of sense of humor on perspective-taking is grounded in the cognitive and social mechanisms of humor processing. First, regarding cognitive processing, humor inherently involves the perception and resolution of incongruity (R. A. Martin, 2007). To comprehend humor, particularly forms such as irony or sarcasm, individuals must process conflicting information and shift from a literal interpretation to an alternative, implied meaning (Skalicky, 2023; Uekermann et al., 2006). This process requires the ability to detach from a singular, egocentric cognitive framework and adopt a different perspective (Filik et al., 2019). Second, regarding social interaction, the effective production of humor requires individuals to tailor their content to the listener’s perspective to ensure comprehension and appreciation (Bitterly et al., 2017). To ensure humor is socially appropriate rather than offensive, individuals should anticipate the mental states, knowledge, and potential emotional reactions of their interlocutors (McGraw & Warren, 2010). Thus, the recurrent practice of formulating and delivering humor facilitates the development of accurate perspective-taking abilities.

By grasping the views of others, individuals can better perceive the origins and nuances of others’ emotions (Cuff et al., 2016). Through the practice of perspective transformation, individuals not only broaden their cognitive range but also enhance their ability to regulate interactions based on this understanding (Galinsky et al., 2005; Gross, 2015). Consequently, this enhanced perspective-taking capacity directly promotes the development of emotional intelligence (Lanning et al., 2023). Integrating these findings with the cognitive mechanisms of humor discussed earlier, a clear mediating pathway emerges. Therefore, we can propose Hypothesis (H3): A sense of humor positively predicts emotional intelligence through the mediating effect of perspective-taking.

Based on the consideration of the relationships among these variables, we further propose hypothesis (H4): Perspective-taking and emotional intelligence jointly act as multiple mediators in the relationship between humor and subjective well-being. Specifically, a sense of humor can improve an individual’s ability of perspective-taking and promote emotional intelligence through the role of perspective-taking. This chain of cognitive and emotional competence helps individuals cultivate a positive mindset and improve interpersonal relationships, ultimately leading to a higher level of subjective well-being. To comprehensively investigate the impact of humor as an independent variable, this study will also examine the four dimensions of humor as independent variables. Given that attitudes towards humor, humor production, coping humor, and humor appreciation all entail emotional comprehension and draw upon social resources, it is posited that each dimension can exert similar effects on perspective-taking, emotional intelligence, and subjective well-being. Thus, the following hypotheses are proposed: the four dimensions of humor (attitude towards humor, humor production, coping humor, and humor appreciation) positively predict subjective well-being. Additionally, perspective-taking and emotional intelligence are hypothesized to act as sequential mediators in the relationship between four dimensions of humor and subjective well-being (H5).

The current study is designed to evaluate the mechanism through which humor impacts subjective well-being and formulates five hypotheses. Firstly, it is hypothesized that a sense of humor can directly forecast the level of subjective well-being (H1). Secondly, it is postulated that emotional intelligence can independently mediate the association between a sense of humor and subjective well-being (H2). Thirdly, perspective-taking acts as a mediator in the relationship between the sense of humor and emotional intelligence (H3). Fourthly, the study examines the chain mediation effect of perspective-taking and emotional intelligence on the relationship between humor and subjective well-being (H4). Finally, an investigation is carried out to determine whether the four dimensions of a sense of humor have an equivalent effect on this mechanism (H5).

### 1.3. The Present Study

**Figure 1:**
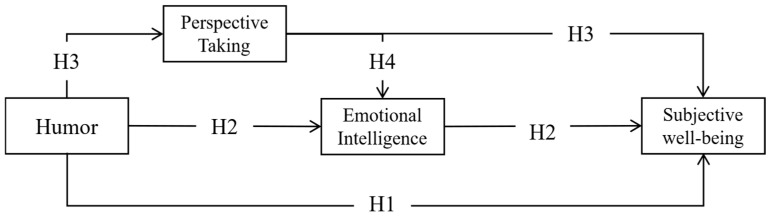
Hypothetical model. The models show the relationship between the main variables. The arrows indicate the mechanism of influence between the variables.

The current study aims to evaluate the mechanism through which humor impacts subjective well-being. Based on the preceding literature review and rationale, five hypotheses are proposed (Figure 1):

**H1.**  *A sense of humor can directly predict the level of subjective well-being.*

**H2.**  *Emotional intelligence acts as a mediator in the association between a sense of humor and subjective well-being.*

**H3.**  *Perspective-taking acts as a mediator in the relationship between the sense of humor and emotional intelligence.*

**H4.**  *Perspective-taking and emotional intelligence exert a serial multiple mediation effect in the relationship between humor and subjective well-being.*

**H5.**  *The four dimensions of a sense of humor will differentially contribute to this proposed mechanism.*

## 2. Materials and Methods

### 2.1. Participants

In this study, a professional platform that is named ‘Wenjuanxing’ was used for the data collection questionnaire survey. From December 2023 to February 2024, a total of 1007 samples were collected after invalid samples were excluded due to catching items that did not pass or the response pattern being unreasonable; thus, the valid response rate was 90.47%. Of the valid samples, 231 and 776 were male and female, respectively. We have obtained informed consent from the subjects or their guardians.

### 2.2. Measures

#### 2.2.1. Multidimensional Sense of Humor Scale (MSHS)

The Multidimensional Sense of Humor Scale was developed by Thorson and Powell (1993). The scale includes 24 items and comprises four dimensions: humor production (11 items), humor appreciation (2 items), coping or adaptive humor (4 items), and attitudes toward humor (7 items). A representative item is, “I use humor to entertain my friends.” Participants responded on a 7-point scale, in which 1 indicates “strongly disagree” and 7 indicates “strongly agree”. The twelfth, fourteenth, fifteenth, sixteenth, eighteenth, and twenty-fourth items are reverse-scored, while the remaining items are scored in the forward direction. Previous research has confirmed its robust construct validity within Chinese populations (Ho et al., 2012). In the current study, the scale demonstrated excellent internal consistency, with a Cronbach’s alpha of 0.928 for the overall scale and ranging from 0.753 to 0.962 for the subscales.

#### 2.2.2. The Subjective Well-Being Questionnaire (SWB)

The Subjective Well-being Questionnaire used in this study was developed by Fosco et al. (2020). The Subjective Well-being Questionnaire consists of 3 items, such as “How much of the time today did you feel happy?” A 7-point scoring scale was used, where 1 represents “strongly disagree”, and 7 represents “strongly agree”. The Chinese version of this scale has demonstrated well-established cross-cultural validity (Li, 2022). In our study, the Cronbach’s alpha for this scale was 0.933.

#### 2.2.3. Perspective-Taking Scale (PT)

The Perspective-Taking Scale was a subscale of the Interpersonal Reactivity Index Scale developed by Davis et al. (1980), which includes 5 items. A representative item is, “When I’m upset at someone, I usually try to put myself in his or her shoes for a while.” A 5-point Likert scale was used (1 = “strongly disagree”, 5 = “strongly agree”). The perspective-taking is presented as each participant’s mean score on the 5 items, with a higher score indicating a higher level of perspective-taking. The Chinese version of the scale has been validated (Qu et al., 2022). In the present study, the Cronbach’s α was 0.863, which is acceptable.

#### 2.2.4. Wong and Law Emotional Intelligence Scale (WLEIS)

The Wong and Law Emotional Intelligence Scale was developed by Wong and Law (2002), according to the emotional theory of Salovey and Mayer (1990), which has been proven to be applicable to Chinese. This scale contains 16 items across four dimensions: self-emotional monitoring (4 items), assessment of others’ emotions (4 items), emotional regulation (4 items), and emotional application (4 items). Participants rated each item on a 5-point Likert scale (1 = “strongly disagree”; 5 = “strongly agree”). A representative item is, “I have a good understanding of my own emotions.” The emotional intelligence score was derived by averaging each participant’s scores on 14 items; the higher the score, the higher the individual’s emotional intelligence. The scale has been widely validated in Chinese contexts (Shi & Wang, 2007). In the present study, the Cronbach’s α of the overall scale was 0.947.

### 2.3. Data Analysis

IBM SPSS 27.0 was employed for preliminary data processing, descriptive statistics, reliability analysis, and correlation analysis between variables. The PROCESS v5.0 macro program (http://www.afhayes.com) (accessed on 19 February 2026) was utilized to conduct mediation analysis, and Model 6 was employed to examine the chain mediation effects of perspective-taking and emotional intelligence in the relationship between sense of humor and subjective well-being.

## 3. Results

### 3.1. Common Method Bias Test

To assess potential common method biases stemming from the self-assessment questionnaire utilized for data collection, the study employed the Harman single-factor test (Harman, 1976). An exploratory factor analysis was conducted on all items comprising the four scales. Results indicated that nine factors possessed eigenvalues greater than 1. However, the first factor explained only 36.09% of the total variance, falling below the 40% threshold criterion proposed by Podsakoff et al. (2003). Consequently, it suggests that common method bias is unlikely to significantly influence the interpretations of the data analysis results.

### 3.2. Descriptive Statistics

The results presented in Table 1 encompass descriptive statistics and correlation analyses among the variables under investigation. Correlation analysis revealed significant positive associations between various constructs. Moreover, each dimension of humor—attitudes toward humor, humor production, coping humor, and humor appreciation—demonstrated significant positive correlations with subjective well-being, perspective-taking, and emotional intelligence, underscoring their potential role in enhancing subjective well-being. Additionally, gender and age variables displayed significant correlations with several constructs, underscoring the importance of including them as control variables in subsequent analyses. Overall, these findings provide valuable insights into the interrelationships among sense of humor, perspective-taking, emotional intelligence, and subjective well-being, laying the groundwork for further investigation into the underlying mechanisms and implications for psychological well-being.

### 3.3. Testing for Chain-Mediated Effects

The study employed Model 6 of the PROCESS macro to investigate the chain mediation effect of perspective-taking and emotional intelligence. Firstly, the chain mediation effect of perspective-taking and emotional intelligence between sense of humor and subjective well-being was examined. Subsequently, the analysis was extended to evaluate the chain mediation effect within each of the four dimensions of humor: attitudes toward humor (AH), humor production (HP), coping humor (CH), and humor appreciation (HA).

Table 2 displays the outcomes of the chain mediation analysis examining the effects of perspective-taking and emotional intelligence between sense of humor and subjective well-being. The findings of this study suggest that sense of humor predicts SWB directly (*B* = 0.216, *t* = 4.471, *p* < 0.001) and has a significant positive effect on perspective-taking (*B* = 0.395, *t* = 18.087, *p* < 0.001) and emotional intelligence (*B* = 0.375, *t* = 11.989, *p* < 0.001). Additionally, perspective-taking has a positive effect on emotional intelligence (*B* = 0.566, *t* = 14.419, *p* < 0.001). Emotional intelligence significantly predicts SWB positively (*B* = 0.598, *t* = 13.099, *p* < 0.001).

**Figure 2:**
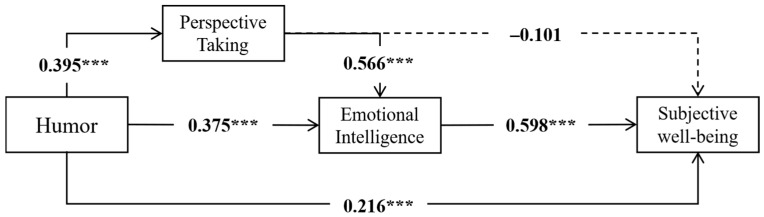
The chain mediation models of humor (N = 1007). The numbers listed in the figure are unstandardized indicators; *** *p* < 0.001.

The mediating path was further tested, and the results showed (see Table 3) that the total effect of humor on SWB was significant (effect = 0.534, 95% CI = [0.450, 0.618]). Furthermore, humor had a significant indirect effect on SWB through emotional intelligence (effect = 0.031, 95% CI = [0.166, 0.287]), accounting for 41.95% of the total effect; however, the indirect effect of humor on SWB through perspective-taking was not significant (effect = −0.040, 95% CI = [−0.099, 0.019]). The serial mediation effect was significant (effect = 0.134, 95% CI = [0.096, 0.177]), accounting for 25.09% of the total effect, which essentially supports our model hypothesis. Based on these results, the chain mediation model depicting the relationship between humor and subjective well-being was constructed, as depicted in Figure 2.

**Figure 3:**
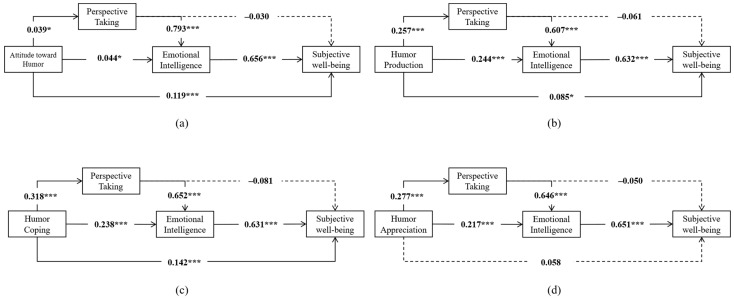
The chain mediation models of humor (N = 1007). In sub-figure (**a**), the independent variable is posited as the attitude toward humor. In (**b**), it is humor production; in (**c**), humor coping; and in (**d**), humor appreciation. The numerals presented in the figure are unstandardized coefficients. The numbers listed in the figure are unstandardized indicators; * *p* < 0.05; *** *p* < 0.001.

Upon further examination (see Appendix A, Table A1, Table A2, Table A3, Table A4, Table A5, Table A6, Table A7 and Table A8), significant chain mediation effects were observed across all dimensions of sense of humor (attitude toward humor, attitude toward humor, humor production, humor coping, and humor appreciation). This indicates that the chain mediation effect of perspective-taking and emotional intelligence in the relationship between humor and subjective well-being is significant, with the confidence interval of the mediation effect excluding a value of 0. Additionally, the mediation effect of perspective-taking between humor and subjective well-being is not significant, as the confidence interval of the mediation effect contains a value of 0. However, the results of direct effects are consistent only across three dimensions of sense of humor: attitude toward humor, humor production, and humor coping. Conversely, the result of humor appreciation differs from that of sense of humor. These findings led to the construction of a chain mediation model illustrating the relationship between sense of humor and subjective well-being, as depicted in Figure 3. To further examine the robustness of our findings, we conducted sensitivity analyses by creating age subgroups based on the sample’s age mean (±1 standard deviation) and re-estimating the same serial mediation model. The results were similar to the main analysis, suggesting that the primary conclusions remained reasonably stable across age subgroups (see Appendix A, Table A9, Table A10, Table A11, Table A12, Table A13, Table A14, Table A15, Table A16, Table A17 and Table A18).

## 4. Discussion

In the Chinese context, this study probed the relationship between sense of humor and subjective well-being and the mechanisms underlying it. The results supported Hypotheses 1–5. The findings show that sense of humor not only directly influences subjective well-being but also indirectly affects it via emotional intelligence. Perspective-taking mediates the link between sense of humor and emotional intelligence. Moreover, perspective-taking and emotional intelligence jointly mediate the relation between sense of humor and subjective well-being. These multiple effects are also significant when the dependent variable is each humor dimension (attitude, production, coping, and appreciation).

Firstly, our study confirmed that sense of humor positively promotes subjective well-being. As a key factor for maintaining well-being and an effective coping style, humor can reduce the decline of positive emotions predicted by the hedonic treadmill model (Lee et al., 2020). Consequently, individuals sustain and even enhance positive emotions after pleasant events. Using humor during negative events also lowers negative emotions such as stress (Morgan et al., 2019), mitigating well-being loss. Therefore, people with a high sense of humor tend to report higher subjective well-being, consistent with prior research (Sousa et al., 2019). As a positive emotional expression, humor’s predictive effect on well-being enriches the well-being framework, highlighting the roles of emotional regulation and social interaction. Practically, the findings suggest new ideas for mental-health interventions; humor can serve as an effective emotion-regulation strategy to improve life satisfaction and mental health under stress.

Secondly, the mediating role of emotional intelligence between sense of humor and subjective well-being was confirmed. Emotional intelligence involves not only managing one’s own emotions but also understanding others’ (Goleman, 1998). Using humor requires acute awareness of personal emotions and context, aiding self-regulation in distress (Yip & Martin, 2006). Humor also enhances interpersonal interaction, fostering rapport (Kim et al., 2022). Hence, individuals with greater humor tend to have higher emotional intelligence, which leads to more stable positive experiences, better relationships, and higher subjective well-being. This provides a new theoretical basis for well-being formation and advances research on emotional regulation, especially humor’s role as a regulation tool. Practically, humor can be employed in interventions to regulate emotions and encouraged across settings to relieve stress, strengthen relationships, and boost overall well-being; for example, workplace job satisfaction can improve by enhancing employees’ humor and emotional intelligence.

Meanwhile, the study reveals that perspective-taking mediates the link between humor and emotional intelligence, and together they serially mediate the effect of sense of humor on subjective well-being. Cultivating humor promotes perspective-taking ability, which raises emotional intelligence and, in turn, elevates subjective well-being. Humor facilitates understanding of varied perspectives, requiring diverse cognitive functions and multiple interpretations (Aykan & Nalçacı, 2018; Shibata et al., 2014). Appreciating humor encourages flexible thinking and receptiveness to different viewpoints (James & Fox, 2024). Perspective-taking, a core component of emotional intelligence, involves understanding and respecting others’ views, emotions, and needs (Ruby & Decety, 2004). By fostering such understanding, individuals improve emotional intelligence, communication, and interpersonal effectiveness (Schröder-Abé & Schütz, 2011). Consequently, those with higher emotional intelligence better manage emotions, communicate, and cope with life’s challenges (Morris, 2023), making them more likely to experience subjective well-being (Llamas-Díaz et al., 2022).

The results from the four dimensions of humor also support the aforementioned conclusions, indicating that Hypothesis 5 is tested. However, only the results from the dimensions of humor attitude, humor production, and coping humor are entirely consistent with those of the humor variable: humor can directly predict subjective well-being, with perspective-taking and emotional intelligence serving as partial mediators. In contrast, the results from the humor appreciation dimension indicate that humor appreciation cannot directly predict subjective well-being. Humor appreciation encompasses understanding the concept of humor, preferences for modes of humor expression, and the degree of acceptance of various types of humor (Suls, 1983). It primarily reflects an individual’s comprehension and perception of humor, assessing humor quality without directly influencing psychological states or behavioral patterns. Consequently, humor appreciation has no direct impact on subjective well-being and is only influenced through the mediating effects of perspective-taking and emotional intelligence. Conversely, utilizing humor as a coping strategy enables individuals to approach life more positively, which is positively correlated with well-being (Oliveira et al., 2023). While humor production includes both the creation and social use of humor, employing humor in social interactions fosters better interpersonal relationships (Thorson & Powell, 1993), which are closely linked to subjective well-being (Zhang et al., 2024). Moreover, individuals with a negative attitude towards humor are less likely to employ it as a coping mechanism or in social communication, and consequently, they exhibit fewer humor-related behaviors.

The investigation into the relationship of perspective-taking, emotional intelligence, sense of humor, and subjective well-being holds significant theoretical and practical implications. Theoretically, this research contributes to a deeper understanding of the relationship among humor, perspective-taking, and emotional intelligence. By exploring the interplay between these factors, it unveils the mechanisms through which humor influences subjective well-being, enriching our comprehension of the relationship between humor and well-being. Furthermore, this study extends the application scope of emotional intelligence theory by linking it to humor and subjective well-being, offering new perspectives and insights for emotional intelligence research. Practically, this research provides specific guidance for interventions in both educational and clinical settings.

In educational contexts, educators can integrate humor-based cognitive training (e.g., interactive improvisational exercises) into social-emotional learning curricula (W. F. Ruch et al., 2018). Compared to interventions requiring specific physical facilities or sustained self-discipline for solitary reflection (Herbert, 2022; Moè, 2022), humor-based activities offer distinct advantages in feasibility and social engagement. Being inherently rewarding and requiring no special equipment, they lower the barrier to entry and foster immediate peer connection, thereby potentially enhancing adherence among university students. By systematically practicing the resolution of humor incongruities, students are guided to step out of egocentric frameworks, thereby enhancing empathy. Clinically, therapists can utilize adaptive humor as a cognitive reappraisal strategy (Samson & Gross, 2012). Guiding clients to view stressful events through a humorous lens facilitates “cognitive decentering,” which is particularly beneficial for reconstructing perspective-taking abilities and emotion regulation in individuals with emotional distress or interpersonal conflicts (Perchtold et al., 2019).

This study has several limitations. First, the research was conducted primarily among young adults in mainland China and covered a relatively narrow age range. Therefore, the findings may not be readily generalizable to other countries or to different populations, such as younger or older individuals and those with clinical conditions or other disabilities. Future studies should extend this work to more diverse cultural contexts, broader age groups, and varied populations to enhance the generalizability of the findings. Second, although the study controlled for some potential confounding variables, there may still be unconsidered factors influencing the relationship between humor and subjective well-being, such as individual personality traits and social support (Mussone & Changizi, 2023). Future research should adopt more rigorous controls. Third, subjective well-being was measured using a three-item measure, which may not fully capture the multidimensional aspects of well-being. Future studies should employ more comprehensive, multidimensional measures of well-being. Finally, the present study adopted a cross-sectional design, which restricts causal inference (Maxwell & Cole, 2007). A bidirectional relationship may exist where individuals with higher subjective well-being experience frequent positive affect, which facilitates humor production (Forgas & Matovic, 2020). Longitudinal research is needed to clarify the directionality and causal nature of these associations.

## 5. Conclusions

Sense of humor positively predicts subjective well-being. Emotional intelligence partially mediates the relationship between sense of humor and subjective well-being, and perspective-taking plays a mediated role in the relationship between sense of humor and emotional intelligence. Additionally, perspective-taking and emotional intelligence play multiple mediated roles in the relationship between sense of humor and subjective well-being, and the model is significantly positive. Furthermore, the model holds true when the independent variables are the three sub-dimensions of humor (attitude toward humor, humor production, and coping humor). However, when the variable is humor appreciation, it only affects subjective well-being through the multiple mediation of perspective-taking and emotional intelligence but cannot directly predict subjective well-being.

## Figures and Tables

**Table 1:** The description results (N = 1007).

Variable	M	SD	1	2	3	4	5	6	7	8	9
1. Sense of humor	4.977	0.837	-								
2. Attitudes toward humor	5.083	1.267	0.473 ***	-							
3. Humor production	4.695	1.166	0.838 ***	0.015	-						
4. Coping humor	5.206	0.935	0.826 ***	0.530 ***	0.453 ***	-					
5. Humor appreciation	5.519	1.071	0.722 ***	0.360 ***	0.452 ***	0.727 ***	-				
6. Perspective-taking	3.723	0.666	0.494 ***	0.068 *	0.451 ***	0.438 ***	0.439 ***	-			
7. Emotional intelligence	4.893	0.947	0.533 ***	0.090 **	0.500 ***	0.439 ***	0.453 ***	0.569 ***	-		
8. Subjective well-being	4.671	1.224	0.371 ***	0.170 ***	0.311 ***	0.309 ***	0.274 ***	0.282 ***	0.516 ***	-	
9. Gender	1.770	0.421	0.040	0.124 ***	−0.039	0.085 **	0.050	−0.043	−0.087 **	−0.018	-
10. Age	23.850	7.740	0.071 *	0.014	0.037	0.096 **	0.122 ***	0.033	0.151 ***	0.129 ***	−0.069 *

Note: * *p* < 0.05, ** *p* < 0.01, *** *p* < 0.001.

**Table 2:** Tests of the chain mediation effect of perspective-taking and intelligence in humor and subjective well-being (N = 1007).

Regression Equation		Overall Fit Index		Regression Coefficient				
Outcome Variable	Predictor Variable	*R* ^2^	*F*	*B*	*SE*	*LLCI*	*ULCI*	*t*
PT	Humor	0.248	110.271	0.395	0.022	0.352	0.438	18.087 ***
EI	Humor	0.426	185.985	0.375	0.031	0.314	0.436	11.989 ***
	PT			0.566	0.039	0.489	0.643	14.419 ***
SWB	Humor	0.284	79.488	0.216	0.048	0.121	0.311	4.471 ***
	PT			−0.101	0.062	−0.224	0.021	−1.628
	EI			0.598	0.046	0.508	0.687	13.099 ***
	Gender			0.052	0.079	−0.102	0.207	0.662
	Age			0.008	0.004	−0.000	0.017	1.890

Note: Standardized indicators are presented; *** *p* < 0.001; PT—perspective-taking, EI—emotional intelligence, SWB—subjective well-being; *LLCI*—lower limit at 95% confidence interval, *ULCI*—upper limit at 95% confidence interval.

**Table 3:** Tests for the chain mediation effect of perspective-taking and emotional intelligence on humor and subjective well-being (N = 1007).

Benefit Type	Path Relationship	Standardized Effect Size	Boot SE	*LLCI*	*ULCI*	Effect Proportion
Total effect		0.534	0.043	0.450	0.618	100.00%
Direct effect	Humor→SWB	0.216	0.048	0.121	0.310	40.45%
Indirect effect 1	Humor→PT→SWB	−0.040	0.030	−0.099	0.019	-
Indirect effect 2	Humor→EI→SWB	0.224	0.031	0.166	0.287	41.95%
Indirect effect 3	Humor→PT→EI→SWB	0.134	0.021	0.096	0.177	25.09%
Total indirect effect		0.318	0.044	0.233	0.405	59.55%

Note: Standardized indicators are presented; PT—perspective-taking, EI—emotional intelligence, SWB—subjective well-being, *LLCI*—lower limit at 95% confidence interval, and *ULCI*—upper limit at 95% confidence interval.

## Data Availability

The datasets generated during and/or analysis during the current study are available in the Dataverse repository, [https://doi.org/10.7910/DVN/99FN8F].

## Abbreviations

PT	Perspective-Taking
EI	Emotional Intelligence
SWB	Subjective Well-being
AH	Attitude toward Humor
HP	Humor Production
HC	Humor coping
HA	Humor Appreciation

## Appendix A

**Table A1:** Tests of the chain mediation effect of perspective-taking and intelligence in attitudes toward humor and subjective well-being (N = 1007).

Regression Equation		Overall Fit Index		Regression Coefficient				
Outcome Variable	Predictor Variable	*R* ^2^	*F*	*B*	*SE*	*LLCI*	*ULCI*	*t*
PT	AH	0.008	2.714	0.039	0.017	0.006	0.071	2.317 *
EI	AH	0.347	133.178	0.044	0.019	0.006	0.081	2.262 *
	PT			0.793	0.036	0.722	0.865	21.764 ***
SWB	AH	0.285	79.634	0.119	0.026	0.067	0.170	4.530 ***
	PT			−0.030	0.060	−0.148	0.088	−0.502
	EI			0.656	0.043	0.572	0.740	15.342 ***
	Gender			0.041	0.079	−0.114	0.196	0.521
	Age			0.008	0.004	−0.000	0.017	1.907

Note: Standardized indicators are presented; * *p* < 0.05, *** *p* < 0.001; AH attitude toward humor, PT perspective-taking, EI emotional intelligence, SWB subjective well-being; *LLCI* = Lower limit at 95% confidence interval, *ULCI* = Upper limit at 95% confidence interval.

**Table A2:** Tests for the chain mediation effect of perspective-taking and emotional intelligence on attitude toward humor and subjective well-being (N = 1007).

Benefit Type	Path Relationship	Standardized Effect Size	Boot SE	*LLCI*	*ULCI*	Effect Proportion
Total effect		0.166	0.030	0.107	0.225	100.00%
Direct effect	AH→SWB	0.119	0.026	0.067	0.170	71.68%
Indirect effect 1	AH→PT→SWB	−0.001	0.003	−0.008	0.005	-
Indirect effect 2	AH→EI→SWB	0.029	0.016	−0.001	0.060	-
Indirect effect 3	AH→PT→EI→SWB	0.020	0.011	−0.001	0.043	-
Total indirect effect		0.048	0.020	0.010	0.088	28.92%

Note: Standardized indicators are presented; AH attitude toward humor, PT perspective-taking, EI emotional intelligence, SWB subjective well-being; *LLCI* = Lower limit at 95% confidence interval, *ULCI* = Upper limit at 95% confidence interval.

**Table A3:** Tests of the chain mediation effect of perspective-taking and intelligence in humor production and subjective well-being (N = 1007).

Regression Equation		Overall Fit Index		Regression Coefficient				
Outcome Variable	Predictor Variable	*R* ^2^	*F*	*B*	*SE*	*LLCI*	*ULCI*	*t*
PT	HP	0.204	85.699	0.257	0.016	0.225	0.288	15.926 ***
EI	HP	0.416	178.253	0.244	0.022	0.201	0.287	11.110 ***
	PT			0.607	0.039	0.532	0.683	15.774 ***
SWB	HP	0.275	75.782	0.085	0.033	0.019	0.151	2.541 *
	PT			−0.061	0.062	−0.183	0.060	−0.988
	EI			0.632	0.046	0.542	0.721	13.875 ***
	Gender			0.088	0.079	−0.066	0.243	1.122
	Age			0.009	0.004	0.000	0.017	2.011 *

Note: Standardized indicators are presented; * *p* < 0.05, *** *p* < 0.001; HP humor production, PT perspective-taking, EI emotional intelligence, SWB subjective well-being; *LLCI* = Lower limit at 95% confidence interval, *ULCI* = Upper limit at 95% confidence interval.

**Table A4:** Tests for the chain mediation effect of perspective-taking and emotional intelligence on humor production and subjective well-being (N = 1007).

Benefit Type	Path Relationship	Standardized Effect Size	Boot SE	*LLCI*	*ULCI*	Effect Proportion
Total effect		0.322	0.031	0.261	0.384	100.00%
Direct effect	HP→SWG	0.085	0.034	0.019	0.151	26.40%
Indirect effect 1	HP→PT→SWB	−0.016	0.019	−0.052	0.021	-
Indirect effect 2	HP→EI→SWB	0.154	0.021	0.115	0.197	47.83%
Indirect effect 3	HP→PT→EI→SWB	0.098	0.014	0.072	0.128	30.43%
Total indirect effect		0.237	0.028	0.184	0.292	73.60%

Note: Standardized indicators are presented; HP humor production, PT perspective-taking, EI emotional intelligence, SWB subjective well-being; *LLCI* = Lower limit at 95% confidence interval, *ULCI* = Upper limit at 95% confidence interval.

**Table A5:** Tests of the chain mediation effect of perspective-taking and intelligence in humor coping and subjective well-being (N = 1007).

Regression Equation		Overall Fit Index		Regression Coefficient				
Outcome Variable	Predictor Variable	*R* ^2^	*F*	*B*	*SE*	*LLCI*	*ULCI*	*t*
PT	HC	0.198	82.700	0.318	0.020	0.278	0.358	15.642 ***
EI	HC	0.387	158.379	0.238	0.028	0.183	0.293	8.442 ***
	PT			0.652	0.039	0.575	0.729	16.613 ***
SWB	HC	0.279	77.286	0.142	0.041	0.061	0.222	3.457 ***
	PT			−0.081	0.062	−0.203	0.042	−1.293
	EI			0.631	0.044	0.544	0.718	14.220 ***
	Gender			0.049	0.079	−0.107	0.205	0.621
	Age			0.008	0.004	−0.001	0.016	1.728

Note: Standardized indicators are presented; *** *p* < 0.001; HC humor coping, PT perspective-taking, EI emotional intelligence, SWB subjective well-being; *LLCI* = Lower limit at 95% confidence interval, *ULCI* = Upper limit at 95% confidence interval.

**Table A6:** Tests for the chain mediation effect of perspective-taking and emotional intelligence on humor coping and subjective well-being (N = 1007).

Benefit Type	Path Relationship	Standardized Effect Size	Boot SE	*LLCI*	*ULCI*	Effect Proportion
Total effect		0.397	0.039	0.319	0.474	100.00%
Direct effect	HC→SWG	0.142	0.041	0.061	0.222	35.77%
Indirect effect 1	HC→PT→SWB	−0.026	0.023	−0.069	0.021	-
Indirect effect 2	HC→EI→SWB	0.150	0.026	0.105	0.204	29.11%
Indirect effect 3	HC→PT→EI→SWB	0.131	0.019	0.095	0.171	24.85%
Total indirect effect		0.255	0.035	0.189	0.323	48.84%

Note: Standardized indicators are presented; HC humor coping, PT perspective-taking, EI emotional intelligence, SWB subjective well-being; *LLCI* = Lower limit at 95% confidence interval, *ULCI* = Upper limit at 95% confidence interval.

**Table A7:** Tests of the chain mediation effect of perspective-taking and intelligence in humor appreciation and subjective well-being (N = 1007).

Regression Equation		Overall Fit Index		Regression Coefficient				
Outcome Variable	Predictor Variable	*R* ^2^	*F*	*B*	*SE*	*LLCI*	*ULCI*	*t*
PT	HA	0.197	82.206	0.277	0.018	0.242	0.312	15.595 ***
EI	HA	0.391	161.028	0.217	0.025	0.169	0.265	8.844 ***
	PT			0.646	0.039	0.570	0.723	16.522 ***
SWB	HA	0.272	74.715	0.058	0.036	−0.013	0.128	1.600
	PT			−0.050	0.062	−0.173	0.073	−0.800
	EI			0.651	0.045	0.563	0.739	14.561 ***
	Gender			0.076	0.079	−0.081	0.231	0.948
	Age			0.008	0.004	−0.001	0.016	1.787

Note: Standardized indicators are presented; *** *p* < 0.001; HA humor appreciation, PT perspective-taking, EI emotional intelligence, SWB subjective well-being. *LLCI* = Lower limit at 95% confidence interval; *ULCI* = Upper limit at 95% confidence interval.

**Table A8:** Tests for the chain mediation effect of perspective-taking and emotional intelligence on humor appreciation and subjective well-being (N = 1007).

Benefit Type	Path Relationship	Standardized Effect Size	Boot SE	*LLCI*	*ULCI*	Effect Proportion
Total effect		0.301	0.035	0.233	0.370	100.00%
Direct effect	HA→SWB	0.058	0.036	−0.013	0.128	-
Indirect effect 1	HA→PT→SWB	−0.014	0.020	−0.053	0.024	-
Indirect effect 2	HA→EI→SWB	0.141	0.022	0.099	0.187	46.84%
Indirect effect 3	HA→PT→EI→SWB	0.117	0.017	0.085	0.151	38.87%
Total indirect effect		0.244	0.030	0.184	0.303	81.06%

Note: Standardized indicators are presented; HA humor appreciation, PT perspective-taking, EI emotional intelligence, SWB subjective well-being; *LLCI* = Lower limit at 95% confidence interval, *ULCI* = Upper limit at 95% confidence interval.

**Table A9:** Tests of the chain mediation effect of perspective-taking and intelligence in humor and subjective well-being (N = 903).

Regression Equation		Overall Fit Index		Regression Coefficient				
Outcome Variable	Predictor Variable	*R* ^2^	*F*	*B*	*SE*	*LLCI*	*ULCI*	*t*
PT	Humor	0.232	90.498	0.381	0.023	0.335	0.426	16.448 ***
EI	Humor	0.390	143.748	0.384	0.033	0.320	0.449	11.709 ***
	PT			0.520	0.042	0.439	0.602	12.531 ***
SWB	Humor	0.279	69.263	0.244	0.051	0.144	0.343	4.803 ***
	PT			−0.149	0.065	−0.277	−0.022	−2.303 *
	EI			0.601	0.048	0.507	0.696	12.490 ***
	Gender			0.026	0.084	−0.138	0.189	0.306
	Age			0.039	0.012	0.016	0.062	3.327 ***

Note: Standardized indicators are presented; * *p* < 0.05, *** *p* < 0.001; PT perspective-taking, EI emotional intelligence, SWB subjective well-being; *LLCI* = Lower limit at 95% confidence interval, *ULCI* = Upper limit at 95% confidence interval.

**Table A10:** Tests for the chain mediation effect of perspective-taking and emotional intelligence on humor and subjective well-being (N = 903).

Benefit Type	Path Relationship	Standardized Effect Size	Boot SE	*LLCI*	*ULCI*
Total effect		0.537	0.045	0.449	0.626
Direct effect	Humor→SWB	0.244	0.051	0.144	0.344
Indirect effect 1	Humor→PT→SWB	−0.057	0.029	−0.113	−0.0001
Indirect effect 2	Humor→EI→SWB	0.231	0.033	0.170	0.298
Indirect effect 3	Humor→PT→EI→SWB	0.119	0.021	0.081	0.162
Total indirect effect		0.293	0.046	0.202	0.384

Note: Standardized indicators are presented; PT perspective-taking, EI emotional intelligence, SWB subjective well-being; *LLCI* = Lower limit at 95% confidence interval, *ULCI* = Upper limit at 95% confidence interval.

**Table A11:** Tests of the chain mediation effect of perspective-taking and intelligence in attitudes toward humor and subjective well-being (N = 903).

Regression Equation		Overall Fit Index		Regression Coefficient				
Outcome Variable	Predictor Variable	*R* ^2^	*F*	*B*	*SE*	*LLCI*	*ULCI*	*t*
PT	AH	0.082	2.031	0.041	0.018	0.006	0.075	2.316 *
EI	AH	0.302	96.886	0.048	0.021	0.007	0.088	2.317 *
	PT			0.767	0.039	0.670	0.824	19.117 ***
SWB	AH	0.273	67.454	0.113	0.028	0.058	0.168	4.047 ***
	PT			−0.070	0.063	−0.193	0.053	−1.121
	EI			0.671	0.045	0.583	0.760	14.873 ***
	Gender			0.027	0.084	−0.138	0.192	0.319
	Age			0.034	0.012	0.011	0.057	2.896 **

Note: Standardized indicators are presented; * *p* < 0.05, ** *p* < 0.01, *** *p* < 0.001; AH attitude toward humor, PT perspective-taking, EI emotional intelligence, SWB subjective well-being; *LLCI* = Lower limit at 95% confidence interval, *ULCI* = Upper limit at 95% confidence interval.

**Table A12:** Tests for the chain mediation effect of perspective-taking and emotional intelligence on attitude toward humor and subjective well-being (N = 903).

Benefit Type	Path Relationship	Standardized Effect Size	Boot SE	*LLCI*	*ULCI*
Total effect		0.163	0.032	0.100	0.226
Direct effect	AH→SWB	0.113	0.028	0.058	0.168
Indirect effect 1	AH→PT→SWB	−0.003	0.004	−0.011	0.003
Indirect effect 2	AH→EI→SWB	0.032	0.018	−0.003	0.067
Indirect effect 3	AH→PT→EI→SWB	0.020	0.012	−0.001	0.044
Total indirect effect		0.050	0.022	0.009	0.094

Note: Standardized indicators are presented; AH attitude toward humor, PT perspective-taking, EI emotional intelligence, SWB subjective well-being; *LLCI* = Lower limit at 95% confidence interval, *ULCI* = Upper limit at 95% confidence interval.

**Table A13:** Tests of the chain mediation effect of perspective-taking and intelligence in humor production and subjective well-being (N = 903).

Regression Equation		Overall Fit Index		Regression Coefficient				
Outcome Variable	Predictor Variable	*R* ^2^	*F*	*B*	*SE*	*LLCI*	*ULCI*	*t*
PT	HP	0.191	70.500	0.250	0.017	0.216	0.283	14.512 ***
EI	HP	0.378	136.405	0.252	0.023	0.207	0.298	10.791 ***
	PT			0.562	0.041	0.482	0.642	13.759 ***
SWB	HP	0.269	65.996	0.118	0.036	0.048	0.189	3.314 **
	PT			−0.114	0.065	−0.241	0.013	−1.763
	EI			0.631	0.048	0.537	0.725	13.164 ***
	Gender			0.070	0.083	−0.094	0.234	0.840
	Age			0.040	0.012	0.017	0.063	3.385 *

Note: Standardized indicators are presented; * *p* < 0.05, ** *p* < 0.01, *** *p* < 0.001; HP humor production, PT perspective-taking, EI emotional intelligence, SWB subjective well-being; *LLCI* = Lower limit at 95% confidence interval, *ULCI* = Upper limit at 95% confidence interval.

**Table A14:** Tests for the chain mediation effect of perspective-taking and emotional intelligence on humor production and subjective well-being (N = 903).

Benefit Type	Path Relationship	Standardized Effect Size	Boot SE	*LLCI*	*ULCI*
Total effect		0.338	0.033	0.273	0.403
Direct effect	HP→SWG	0.118	0.036	0.048	0.189
Indirect effect 1	HP→PT→SWB	−0.028	0.019	−0.064	0.009
Indirect effect 2	HP→EI→SWB	0.159	0.023	0.117	0.207
Indirect effect 3	HP→PT→EI→SWB	0.089	0.015	0.061	0.120
Total indirect effect		0.219	0.031	0.160	0.279

Note: Standardized indicators are presented; HP humor production, PT perspective-taking, EI emotional intelligence, SWB subjective well-being; *LLCI* = Lower limit at 95% confidence interval, *ULCI* = Upper limit at 95% confidence interval.

**Table A15:** Tests of the chain mediation effect of perspective-taking and intelligence in humor coping and subjective well-being (N = 903).

Regression Equation		Overall Fit Index		Regression Coefficient				
Outcome Variable	Predictor Variable	*R* ^2^	*F*	*B*	*SE*	*LLCI*	*ULCI*	*t*
PT	HC	0.189	69.671	0.310	0.022	0.268	0.352	14.426 ***
EI	HC	0.351	121.501	0.257	0.030	0.199	0.316	8.635 ***
	PT			0.598	0.042	0.516	0.680	14.351 ***
SWB	HC	0.270	66.232	0.150	0.044	0.065	0.236	3.443 ***
	PT			−0.122	0.065	−0.249	0.006	−1.873
	EI			0.640	0.047	0.548	0.733	13.645 ***
	Gender			0.026	0.085	−0.140	0.192	0.309
	Age			0.037	0.012	0.014	0.060	3.108

Note: Standardized indicators are presented; *** *p* < 0.001; HC humor coping, PT perspective-taking, EI emotional intelligence, SWB subjective well-being; *LLCI* = Lower limit at 95% confidence interval, *ULCI* = Upper limit at 95% confidence interval.

**Table A16:** Tests for the chain mediation effect of perspective-taking and emotional intelligence on humor coping and subjective well-being (N = 903).

Benefit Type	Path Relationship	Standardized Effect Size	Boot SE	*LLCI*	*ULCI*
Total effect		0.396	0.042	0.314	0.478
Direct effect	HC→SWG	0.150	0.044	0.065	0.236
Indirect effect 1	HC→PT→SWB	−0.038	0.024	−0.083	0.010
Indirect effect 2	HC→EI→SWB	0.165	0.028	0.112	0.221
Indirect effect 3	HC→PT→EI→SWB	0.119	0.020	0.083	0.162
Total indirect effect		0.246	0.037	0.174	0.320

Note: Standardized indicators are presented; HC humor coping, PT perspective-taking, EI emotional intelligence, SWB subjective well-being; *LLCI* = Lower limit at 95% confidence interval, *ULCI* = Upper limit at 95% confidence interval.

**Table A17:** Tests of the chain mediation effect of perspective-taking and intelligence in humor appreciation and subjective well-being (N = 903).

Regression Equation		Overall Fit Index		Regression Coefficient				
Outcome Variable	Predictor Variable	*R* ^2^	*F*	*B*	*SE*	*LLCI*	*ULCI*	*t*
PT	HA	0.189	69.665	0.268	0.019	0.232	0.305	14.426 ***
EI	HA	0.350	121.000	0.221	0.026	0.170	0.271	8.553 ***
	PT			0.599	0.042	0.518	0.681	13.374 ***
SWB	HA	0.262	63.837	0.066	0.038	−0.009	0.140	1.730
	PT			−0.094	0.065	−0.222	0.035	−1.431
	EI			0.663	0.047	0.570	0.755	14.063 ***
	Gender			0.055	0.085	−0.110	0.221	0.656
	Age			0.036	0.012	0.013	0.059	3.036 **

Note: Standardized indicators are presented; ** *p* < 0.01, *** *p* < 0.001; HA humor appreciation, PT perspective-taking, EI emotional intelligence, SWB subjective well-being. *LLCI* = Lower limit at 95% confidence interval; *ULCI* = Upper limit at 95% confidence interval.

**Table A18:** Tests for the chain mediation effect of perspective-taking and emotional intelligence on humor appreciation and subjective well-being (N = 903).

Benefit Type	Path Relationship	Standardized Effect Size	Boot SE	*LLCI*	*ULCI*
Total effect		0.293	0.037	0.221	0.365
Direct effect	HA→SWB	0.066	0.038	−0.009	0.140
Indirect effect 1	HA→PT→SWB	−0.025	0.020	−0.063	0.015
Indirect effect 2	HA→EI→SWB	0.146	0.024	0.101	0.196
Indirect effect 3	HA→PT→EI→SWB	0.107	0.017	0.075	0.141
Total indirect effect		0.228	0.031	0.168	0.291

Note: Standardized indicators are presented; HA humor appreciation, PT perspective-taking, EI emotional intelligence, SWB subjective well-being; *LLCI* = Lower limit at 95% confidence interval, *ULCI* = Upper limit at 95% confidence interval.
